# Pituitary Microsomal Autoantibodies in Patients with Childhood-Onset Combined Pituitary Hormone Deficiency: an Antigen Identification Attempt

**DOI:** 10.1007/s00005-016-0386-x

**Published:** 2016-03-12

**Authors:** Katarzyna Ziemnicka, Paweł Gut, Monika Gołąb, Grzegorz Dworacki, Elżbieta Wrotkowska, Marek Stajgis, Katarzyna Katulska, Barbara Rabska-Pietrzak, Monika Obara-Moszyńska, Marek Niedziela, Bartłomiej Budny, Małgorzata Kałużna, Ryszard Waśko, Marek Ruchała

**Affiliations:** 1 Chair and Department of Endocrinology, Metabolism and Internal Diseases, Poznan University of Medical Sciences, Poznan, Poland; 2 Chair of Clinical Immunology, Department of Immunology, Poznan University of Medical Sciences, Poznan, Poland; 3 Department of General Radiology and Neuroradiology, Poznan University of Medical Sciences, Poznan, Poland; 4 Department of Pediatric Endocrinology and Rheumatology, Poznan University of Medical Sciences, Poznan, Poland

**Keywords:** Anti-microsomal antibodies, Combined pituitary hormone deficiency, Immunoblotting, Radioimmunology

## Abstract

The role of autoimmunization in the pathogenesis of pituitary disorders is poorly understood. The presence of pituitary autoantibodies (APA) has been detected in various pituitary disorders. Their role, however, remains elusive. Childhood-onset combined pituitary hormone deficiency (CPHD) may be caused by environmental or genetic factors. In some of patients, causes of the disease remain unclear and contributions of autoimmune processes have been postulated. The aim of this study was to identify the microsomes-derived pituitary antigens (MPA) as potential immunogenic autoantigens in patients with hypopituitarism, therefore 62 CPHD patients, 100 healthy controls and five autoimmune polyglandular syndrome type II (APS II) patients were included in the study. The clinical evaluation included hormonal tests and magnetic resonance imaging of the pituitary. The sources of MPA were pituitary glands taken from autopsies. Isolated MPA were then separated on SDS-PAGE gel and incubated with sera obtained from patients and controls. Microsomal APA were detected using Western blot and radioimmunological method. In all CPHD and APS II patients and in 9 % individuals from control group marked immunoreactivity was detected against MPA. Antibodies showed high affinity to 67, 60, 50 and 36 kDa MPAs. Since the identified autoantigens were of unknown nature, an in silico exploration of UniProt database was applied and indicated their possible relationship with chaperones, golgins and already known autoantigens like GAD67. Reactivity against MPA indicates that these proteins certainly play a role in the processes undergoing within pituitary of CPHD patients. The identification and further detailed studies on their role in the pathogenesis of CPHD should be continued.

## Introduction

Combined pituitary hormone deficiency (CPHD) with a childhood-onset is a rare disorder that develops as a consequence of congenital malformations, inflammation, blood flow disturbances or tumours. However, in approximately 10–11 % of patients, the aetiology of the disease remains unknown (Regal et al. 2001). Intensive studies of the pituitary organogenesis revealed an important role of mutations in transcription factor genes, particularly in familial cases of CPHD, what may explain their role in 11.2 % of sporadic cases and in 63 % of familial cases (De Rienzo et al. 2015). However, for a significant number of sporadic patients with early disease onset, the cause of the disease remains unclear. Hence, a contribution of autoimmune processes has been suggested.

The model of autoimmunity affecting the pituitary is lymphocytic hypophysitis (LYH). Most typical signs and symptoms of LYH include pituitary enlargement caused by inflammatory infiltrate and oedema, visual field limitation and hormonal disturbances (Ezzat and Josse 1997; Goudie and Pinkerton 1962; Gutenberg et al. 2006). With the disease progression, due to pituitary destruction and withdrawal of inflammatory infiltrate, the mass of the gland decreases. A partial or complete deficiency of the anterior pituitary hormones as a first symptom may appear in approximately 60 % of patients (Thodou et al. 1995). Up to 15 % LYH patients develop persistent CPHD (Herold and Sarne 2000) and about 20 % of patients have another autoimmune disorder—in most cases chronic lymphocytic thyroiditis (Thodou et al. 1995). Besides LYH, there are probably other symptoms of autoimmunization against the pituitary. De Jersey et al. (2002) created a model of autoimmune reaction against pituitary in transgenic mice expressing influenza virus nucleoprotein (containing epitopes recognized by specific CD8 T cells) under control of the human growth hormone (hGH) promoter. These mice suffer from a destruction of somatotropes, and have a reduced secretion of the growth hormone (GH) and a dwarf phenotype. In parallel to GH deficiency, reduced levels of thyrotropin (TSH) and prolactin (PRL) have also been observed (de Jersey et al. 2002). Several attempts have focused on the detection of the pituitary autoantigens and anti-pituitary autoantibodies. Already known autoantigens contributing to autoimmune hypophysitis include GH, α- and γ-enolase, pituitary gland specific factor 1a and 2, secretogranin II (Bensing et al. 2007a, b) or transcription factors taking part in pituitary cell lines differentiation like TPIT or PIT-1, although, their role remains elusive (Bensing et al. 2005; Nakahara et al. 2005; O’Dwyer et al. 2002; Tanaka et al. 2002, 2003).

The microsomal autoantibodies have been shown to act as pathogenic factors in different autoimmune disorders, like Hashimoto thyroiditis or autoimmune hepatitis (Kasagi et al. 1996; Rizzetto et al. 1973). Therefore, the aim of this study was to identify and to define the role of microsomes-derived autoantigens as potential immunogenic factors, which may trigger the production of pituitary-specific autoantibodies in patients with childhood-onset CPHD.

## Materials and Methods

### Study Subjects

Study group consisted of 62 adults originally diagnosed with childhood-onset CPHD in endocrinology departments having no history of any serious health problems or relevant accidents at delivery or in childhood, which may affect pituitary function (including pituitary tumours, infections, or haematological problems). There were also no signs or symptoms of diabetes insipidus. Seventeen of these patients had been previously treated with rhGH and in 23 patients using bi-directional conventional sequencing *PROP1* gene mutation was revealed (data not shown).

Controls comprised 100 healthy volunteers, without any proven endocrine or autoimmune disorders and no recent drug treatments. Sera from five patients diagnosed with autoimmune polyglandular syndrome type II (APS II) with a previous record of the microsomal anti-pituitary autoantibodies confirmed by the same methodology (Gut et al. 2008) were included as a positive control. All APS II patients were diagnosed with Addison’s disease and autoimmune thyroid disorder and two of them also suffer from vitiligo. Demographic data are presented in Table 1.

**Table 1:** The characteristics of the study subjects: patients with the combined pituitary hormone deficiency (CPHD) and healthy controls

Variable	CPHD patients (*n* = 62)	Healthy controls (*n* = 100)
Sex: *n* (%)		
Males	33 (53.2 %)	55 (55 %)
Females	29 (46.8 %)	45 (45 %)
Age (years): mean ± SD (range)		
At CPHD diagnosis	8.7 ± 7.0 (2–26)	NA
At the time of the immunological study	35.5 ± 14.3 (18–60)	36.9 ± 10.2 (19–60)
CPHD duration time (years)^a^: mean ± SD (range)	26.5 ± 13.5 (6–64)	NA
Pituitary morphology in MRI: *n* (%)		
Hypoplasia of the anterior lobe	40 (64.5 %)	NA
Hypoplasia of the anterior lobe plus pituitary stalk	14 (22.6 %)	NA
Interruption plus ectopy of the posterior pituitary lobe	3 (4.8 %)	NA
Hypoplasia of anterior lobe plus cystic lesions normal pituitary	3 (4.8 %)	NA
Hyper-intensive signal from all pituitary	2 (3.2 %)	NA
Type of pituitary hormone deficiency: *n* (%)		
GH/LH/FSH/TSH	23 (30.1 %)	NA
GH/LH/FSH/TSH/PRL	18 (29.0 %)	NA
GH/LH/FSH/TSH/ACTH	14 (22.6 %)	NA
GH/LH/FSH/TSH/PRL/ACTH	7 (11.3 %)	NA

*NA* not applicable

^a^Approximate duration time of CPHD calculated from the moment of diagnosis

A written informed consent was obtained from all participants and the Bioethical Committee of the Poznan University of Medical Sciences, approved the study. Demographic data are presented in Table 1.

### Evaluation of the Pituitary Morphology and Function in CPHD Patients

The morphology of the pituitary gland was evaluated by magnetic resonance imaging (MRI) with Siemens 1T Magnetom Impact using multislice spin-echo pulse sequences. Sagittal T1-weighted images were acquired with parameters of 800/15/4 (TR/TE/excitations), 3-mm slice thickness with 1 mm interslice gap, 256 × 256 acquisition matrix, and a 24-cm field of view. Coronal T1-weighted images were also obtained with a field of view of 20 cm.

Basal and stimulated levels of the pituitary (GH, TSH, LH/FSH, PRL, and ACTH) and relevant peripheral hormones (IGF-1, fT3, fT4, cortisol and estradiol/testosterone) were measured to assess the pituitary function at the moment of diagnosis and during follow up. The measurements were taken using the following methods: the radioimmunological (RIA) method (Spectria, Orion Diagnostica (Finland) for the GH, the SM-C-RIA-CT kit (BioSource, Belgium) for the IGF-1, and the immunochemiluminometric method (ICMA kits—Elecsys 2010, Roche Diagnostics, Switzerland) for adrenocorticotropin (ACTH), cortisol, TSH and PRL, fT4, lutropin (LH), and follitropin (FSH). Stimulation tests were performed prior to immunological study according to the following procedures: GH response and pituitary-adrenal axis was assessed during insulin-induced hypoglycaemia (0.1 IU/kg; Actrapid Insulin, Novo Nordisk, Denmark) and blood was collected at 0, 15, 30, 45, 60, 90 and 120 min after insulin administration with parallel estimation of glucose concentration. Severe GH deficiency was diagnosed when its concentration did not raise over 3 ng/ml. In the same test cortisol concentration should increase over 500 nmol/l. TSH and prolactin release were estimated in the thyrotropin-releasing hormone stimulation test (200 μg Protirelin i.v. Merck, Germany) and the blood samples were collected at 0, 30, 60 min expecting increase in TSH concentration for at least 2 mIU/l after 30 min and at least double increase in PRL concentration. LH/FSH response was investigated using GnRH test (100 μg GnRH, Ferring, Germany). The expected concentrations of LH were at least 2–3 times higher than its basal level and in the case of FSH—approximately 1.5–2 times higher concentration than the basal level (Larsen et al. 2003). These tests were done at the time of diagnosis and some of them were repeated during follow up.

### Immunological Studies

Serum samples from CPHD cases, controls and APS II patients were tested for the presence of anti-pituitary autoantibodies with a solid-phase RIA method using the microsomal fraction of the human pituitary gland as a source of antigens (MPA: microsomal pituitary antigens). Pituitary glands with no morphological signs of autolytic changes were obtained from autopsy 24 h after death. A pathological verification was conducted on histological slides using standard light microscopy after haematoxylin–eosin staining. Only tissues showing no severe signs of autolysis were processed for further analysis. Remained freshly obtained tissue samples were then frozen down in nitrogen vapours and stored at −70 °C until use. To isolate the antigens, the tissue was homogenised after thawing in 0.05 M phosphate-buffered saline with phenylmethylsulphonylfluoride (PMSF) in the buffer to tissue ratio of 4:1, with protease inhibitors (0.5 ml/10 g of tissue). Afterwards, it was centrifuged for 10 min at 15,000×*g* at 4 °C. The supernatant was centrifuged for 60 min at 105,000×*g* and decanted. The pellet was then resuspended in phosphate-buffered saline (PBS) as before and centrifuged at 105,000×*g* for 60 min. This procedure was repeated twice. The microsomal fraction present in the pellet was resuspended in PBS and solubilised in 0.1 % sodium deoxycholate solution. The protein concentration was estimated spectrophotometrically based on the optical density at 280 nm. Microsomal fraction was stored at −70 °C until use. Staphylococcus protein A (Amersham BioSciences-GE Healthcare) was labelled with ^125^I using chloramine procedure with 50 μg of protein A in phosphorus 0.5 M buffer, pH 7.4, 1 mCi of ^125^I and 25 μg of chloramine. Iodination reaction was stopped after 10 s by adding 60 μg of sodium pirosulfatein, 50 μl PBS buffer and 20 μl of 5 % foetal bovine albumin. Iodinated mixture was transferred to g-25 *fine* Sephadex 1 × 10 cm column and eluted with 0.1 M PBS buffer. One mililiter of fraction from each sample was collected. The first radioactivity fraction contained iodinated ^125^I protein A, the second one—unbound ^125^I. Anti-pituitary autoantibodies were detected in the sera using a solid-phase RIA method. The reaction was conducted in polyethylene tubes coated with proteins from the human pituitary microsomal fraction. The tubes were coated with 3–4 µg of microsomal proteins suspended in 0.5 ml PBS buffer, after prior solubilization in 0.1 % deoxycholate (DOC). After 1-h incubation, 3 % of non-fat milk in PBS was added to block free binding sites. Diluted sera from CPHD patients and controls (1:1000 in physiological salt solution) were subsequently added to the tubes and incubated for 24 h at +4 °C. Afterwards, the content was removed and the tubes were rinsed with PBS. Protein A labelled with ^125^I (activity of 50,000–100,000 cpm) was added to detect bound autoantibodies as described elsewhere (Gryczynska and Kosowicz 1987). The means of the signal intensity in counts per minute were subsequently calculated per each group and presented as percentage of binding per total binding (%B/TB). The mean value obtained for the controls was used as a reference. For each patient, the binding was then classified as low, weakly positive (result exceeding 2SD–3SD of the arithmetic mean for the controls), moderately positive (3SD–4SD of the mean for the controls), or highly positive (over 4SD).

SDS-PAGE electrophoresis was performed on polyacrylamide gel (12.5 % running gel and 6 % stacking gel) under reducing conditions. Microsomal proteins separated by gel electrophoresis were afterwards transferred onto a nitrocellulose membrane using a Western blot electrotransfer buffer (pH 8.3 16 h at 8–10 V) containing Tris–HCl, glycine and methanol (Towbin Buffer, Bio-Rad, USA). A molecular weight marker (Low Molecular Weight Marker; Amersham-Pharmacia) containing a mixture of proteins ranging 14,000–97,000 kDa was used as a reference in each experiment. Afterwards, the nitrocellulose membrane was rinsed in distilled water and stained with Poinceau S to visualize protein bands. Blocking with 3–5 % non-fat milk in Tris-buffered saline (TBS) was followed by incubation with diluted sera (1:200) at 4 °C for 16 h. After the incubation, the nitrocellulose strips were washed in 0.05 % Tween/TBS and TBS buffers. Then, the membranes were incubated with the secondary antibody: rabbit anti-human IgG conjugated with horseradish peroxidase (Sigma-Aldrich, USA), diluted at 1:10,000, for 1 h at room temperature. After the incubation, the membranes were rinsed with TBS-Tween and TBS buffers and subjected to chemiluminescence reaction with a luminol-based substrate for the horseradish peroxidase (Amersham-Pharmacia Biotech, Sweden), followed by autoradiography. We explored UniProt Knowledgebase (UniProtKB Release 2013_12, http://www.uniprot.org) for the mapping of protein isolated from human pituitary microsomes. UniProt database represents a collection of records for proteins extracted from literature and curator-evaluated computational analysis. This proteomic information includes accurate and consistent functional and structural annotations, as well as taxonomic data that are regularly updated. Corresponding proteins to these detected by immunoblots in our study were inferred based on the estimated molecular weight and tissue specificity.

### Tissue Specificity

Sera from ten patients showing highly positive binding of microsomal APA (M-APA), all APS II patients and ten controls were tested using Western blot and RIA. The reactivity against microsomal antigens derived from the thyroid gland was studied. The fresh thyroid for this study was obtained during surgery from patient with simple nodular goitre without signs and symptoms of any autoimmune disorder. The written informed consent was obtained from the patient. Preparation of microsomal antigens was conducted according to the same procedure, as described for the pituitary specimen. RIA method was used to determine the high, moderate and low binding affinity of microsomal thyroid autoantibodies.

### Other Autoantibodies Screening

Evaluation of anti-thyroid peroxidase (TPO) (ICMA; Elecsys 2010, Roche Diagnostics, Switzerland; normal range <34 IU/ml), anti-Tg (ICMA; Elecsys 2010, Roche Diagnostics, Switzerland; normal range <115 IU/ml) and anti-GAD autoantibodies (IRMA; Beckman-Coulter, Immunotech Chech; normal range <1.0 U/ml) was conducted in all studied cases (CPHD, APS II and controls).

### Statistical Analysis

The results were analysed using *χ* ^2^ test with Yates’ correction, unpaired *t* test with Welch correction, and Mann–Whitney test. *p* values <0.05 were considered as indicative of a statistical significance.

## Results

Basic characteristics of the combined CPHD patients and healthy controls as well as the pituitary morphology and function in the CPHD patients are shown in Table 1. Age and sex for the two studied groups (patients and controls) matched. Stimulation tests evaluating pituitary hormone excretion revealed severe deficiencies of GH, TSH, and LH/FSH in all patients. The levels of PRL and ACTH were highly variable. In all patients, IGF-1, fT4, and oestrogen or testosterone levels were below normal range at the moment of diagnosis. The major pituitary hormone deficiency diagnosed in this group was a GH deficit resulting in a marked growth deficiency (92 % of patients). Five patients presented hypothyroidism caused by TSH deficit as the first symptom. MRI of the pituitary showed anterior lobe hypoplasia with or without posterior lobe ectopy in most of CPHD patients (Table 1).

Antibody levels were determined by a solid-phase RIA assay. Taking into considerations only M-APA levels over 2SD, all CPHD and APS II patients had detectable levels of the pituitary anti-microsomal autoantibodies. Among healthy controls, autoantibodies with the level over 2SD were detected only in 9 out of 100 individuals (9 %). The other controls presented very low or undetectable M-APA levels, what was confirmed in Western blot and RIA method. The range of non-specific binding was 0.6–3.1 % of total radioactivity (mean 1.93 %). The mean %B/TB in all controls was 5.4 ± 1.1. Anti-microsomal autoantibodies with a high binding affinity (over 4SD) were detected in 24.2 % of CPHD patients (15/62; mean: %B/TB was 18.1 ± 5.2; range 10.2–26.8 %), 100 % of APS type II patients (5/5; mean:  %B/TB was 25.3 ± 5.4; range 19.8–32 %) and in one healthy control (1/100; 13.4 % B/TB). Moderate binding (3SD-4SD) was detected in 43.5 % of CPHD patients (27/62; mean: %B/TB was 9.3 ± 0.3). Low binding (2SD–3SD) was detected in 32.3 % of CPHD patients (20/62; mean: %B/TB was 8.1 ± 0.4) and in 8 % of the controls (8/100; mean: %B/TB was 7.9 ± 0.3).

**Fig. 1:**
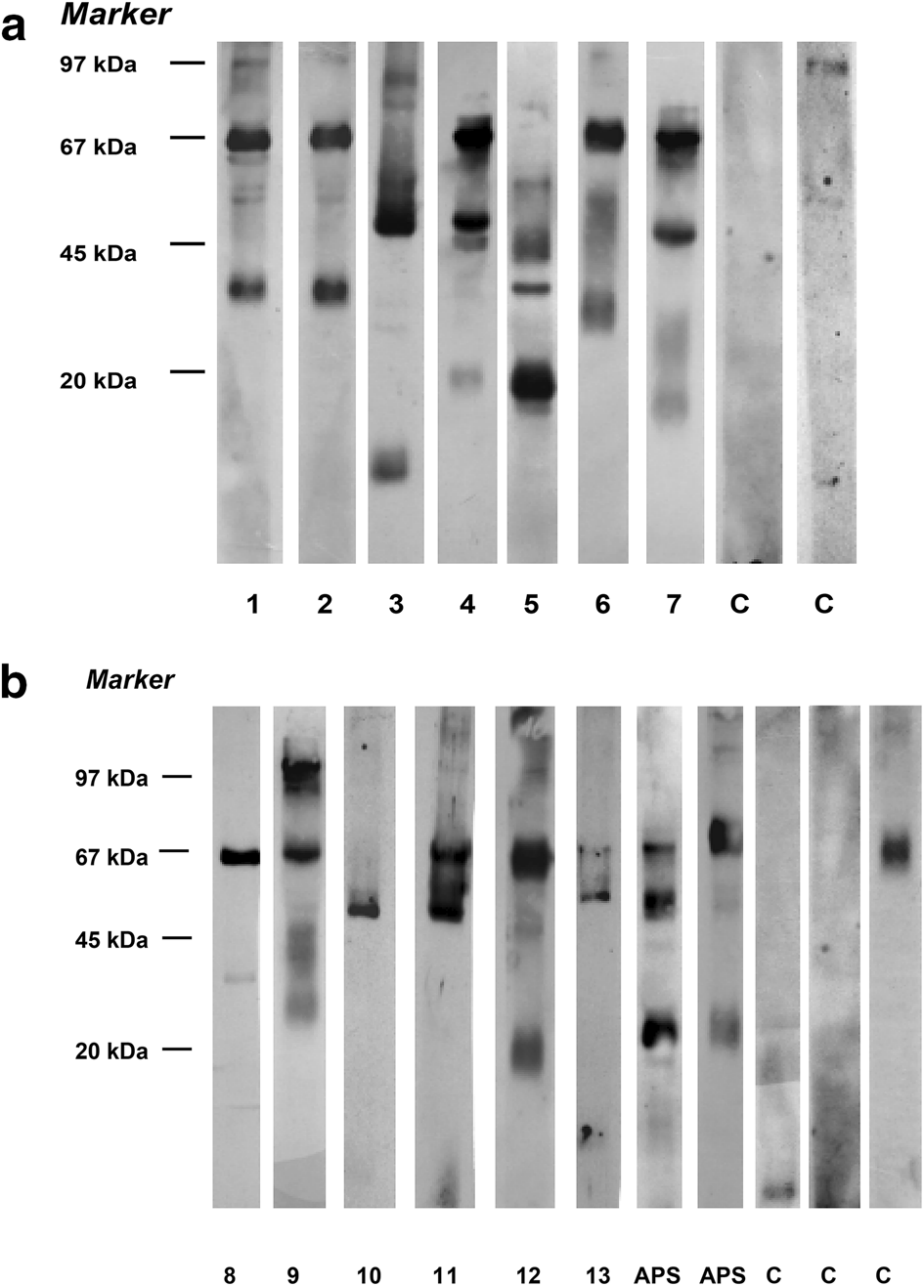
Reactivity of microsomal pituitary antigens (MPA) with autoantibodies present in the sera of the combined pituitary hormone deficiency (CPHD) patients (*lane* 1–13), and healthy controls (C) detected by immunoblotting. **a**, **b** Two different immunoblot’s assays. Sera from type 2 autoimmune polyglandular syndrome patients were used as a positive control (APS) Marker: Low Molecular Weight Marker (Amersham-Pharmacia Biotech, Sweden)

The reactivity targeted mainly the antigens of the following approximate molecular weights: 67 kDa (69.4 % CPHD patients versus 8 % of controls), 55 kDa (50 % versus 4 %), and 36 kDa (22.6 vs. 4 %; Table 2). In APS II patients, auto-reactivity against 67, 60, 55, 36 and 20 kDa autoantigens was showed. Figure 1 shows examples of immunoblots with the use of sera from CPHD and APS II patients, and from the controls. Reactivity against 1–2 antigens was showed in 36 patients (58.1 %), reactivity against three and more antigens was present in 27 patients (49.6 %).

**Table 2:** Reactivity against microsomal antigens isolated from human pituitary gland detected in the sera of combined pituitary hormone deficiency (CPHD) patients and healthy controls using immunoblotting

Microsomal pituitary autoantigen, approximate molecular weight (kDa)	CPHD patients (*n* = 62)		Healthy controls (*n* = 100)	
	*n* positive	% positive	*n* positive	% positive
105	2	3.3	2	2
97	8	12.9	2	2
68	2	3.3	–	–
67	43	69.4	8	8
62	1	2.0	–	–
60	19	30.6	–	–
57	3	4.8	–	–
55	31	50.0	4	4
52	1	2.0	–	–
45	2	4.0	–	–
36	14	22.6	4	4
20	1	2.0	–	–
18	3	6.0	–	–

Patients were divided into groups according to M-APA level (low, moderate, high) and according to number of autoantigens (MPA) recognized by microsomal pituitary autoantibodies. The results of statistical analysis are demonstrated in Tables 3 and 4, respectively.

**Table 3:** Analysis of 62 CPHD patients with high, moderate and low concentration of pituitary microsomal autoantibodies (M-APA)

Variable	CPHD patients with high M-APA level	CPHD patients with moderate M-APA level	CPHD patients with low M-APA level
Column	A	B	C
Total number	15	27	20
Males/females	7/8 (46.7/53.3 %)	15/12 (55.5/44.5 %)	11/9 (55.0/45.0 %)
Duration of CPHD (mean ± SD)	28.5 ± 15.1^a^	31.0 ± 15.6^a^	19.5 ± 8.7^a^
AITD	2 (13.3 %)	2 (7.4 %)	1 (5.0 %)
Prolactin deficiency	7 (46.7 %)	13 (48.1 %)	5 (25.0 %)
ACTH deficiency	8 (53.3 %)	7 (25.9 %)	6 (30.0 %)
MPAs detected by M-APA			
1–2 Ag	8 (53.3 %)	10 (37.0 %)	20 (100 %)
At least 3 Ag	7 (46.7 %)	17 (63.0 %)	0
No. of patients treated with rhGH	5 (33.3 %)	8 (29.6 %)	4 (20.0 %)
Pituitary morphology other than pituitary hypoplasia only	6 (40.0 %)	10 (37.0 %)	6 (30.0 %)

*AITD* autoimmune thyroid disease

^a^Approximate duration time of CPHD calculated from the moment of diagnosis

**Table 4:** Analysis of 62 CPHD patients with reactivity to different number of microsomal pituitary antigen (MPA)

Variable	CPHD patients with reactivity against 1–2 MPA	CPHD patients with reactivity against 3 and more MPA
Total number	36	26
Males/females	19/17 (52.8/47.3 %)	14/12 (53.8/46.2 %)
Duration of CPHD (mean ± SD)	23.9 ± 14.1 years	30.6 ± 13.3 years
AITD	1 (2.8 %)	4 (15.4 %)
Prolactin deficiency	15 (41.7 %)	10 (38.5 %)
ACTH deficiency	11 (30.9 %)	10 (38.5 %)
M-APA level		
High	8 (22.2 %)	7 (26.9 %)
Moderate	10 (27.8 %)	17 (65.4 %)
Low	18 (50.0 %)	2 (7.7 %)
No. of patients treated with rhGH	8 (22.2 %)	9 (34.6 %)
Pituitary morphology other than pituitary hypoplasia only	7 (19.4 %)	13 (50.0 %)

*AITD* autoimmune thyroid disease

Statistical analysis (*χ* ^2^ test with Yates’ correction) did not show any significant differences between patients treated with rhGH and non-treated patients regarding antibodies binding (*p* = 0.640) and number of autoantigens detected by Western blot (*p* = 0.912). The sera of the two patients showing a high intensity pituitary signal in MRI were positive for the presence of anti-pituitary autoantibodies (high level in one patient and moderate level in the other), with reactivity against 67, 55 and 36 kDa proteins.

There was significant difference between patients with reactivity to 1–2 MPA and patients with 3–4 MPA (*χ* ^2^ test with Yates’ correction; *p* = 0.01) regarding pituitary morphology. Patients with more diverse pituitary in MRI study (cysts, stalk interruption etc.) demonstrated immunoreactivity to three or more pituitary antigens.

The presence of *PROP1* gene mutations affected neither clinical signs nor reactivity against MPA (*p* > 0.05), there was also no difference between patients presented prolactin and ACTH deficiency and those who did not.

### Other Autoantibodies

Anti-TPO antibodies were positive only in five patients with CPHD (range 56–128 IU/ml) and in all patients with APS II (over 2000 IU/ml). Anti-Tg antibodies were positive in one CPHD patients (with elevated anti-TPO; 120 IU/ml) and in all patients with APS II (over 500 IU/ml). Both antithyroid antibodies were negative in the control group. Anti-GAD antibodies were positive in two patients with APS II (1.1 and 1.5 U/ml) but negative in all subjects from CPHD group and in the controls.

### Characteristics of Patients with High Level of Microsomal Anti-Pituitary Antibodies

Sera from 15 CPHD (eight female and seven male) patients showed high binding affinity to MPA. In all these patients reactivity to 67 kDa autoantigen was found, the next most common reactivity was presented against 55 and 36 kDa. Two of them had also high level of anti-TPO and anti-Tg antibodies. The mean duration of CPHD in this group was 28.46 ± 15.13 years, what statistically differs from the group with moderate (unpaired *t* test with Welch correction; *p* = 0.0005) and low binding (*p* = 0.03). Reactivity against three or more autoantigens was found in 8 out of 15 patients (53.33 %). Number of detected autoantigens also increased with age (Mann–Whitney test; *p* = 0.04). Most common finding on the MRI study was pituitary hypoplasia that was present in eight patients, in the others in addition to hypoplasia; pituitary cyst or pituitary stalk interruption syndrome (PSIS) were detected. In more than 50 % of patients ACTH and prolactin deficiencies were demonstrated. Detailed data are presented in Table 3.

### Tissue Specificity

**Fig. 2:**
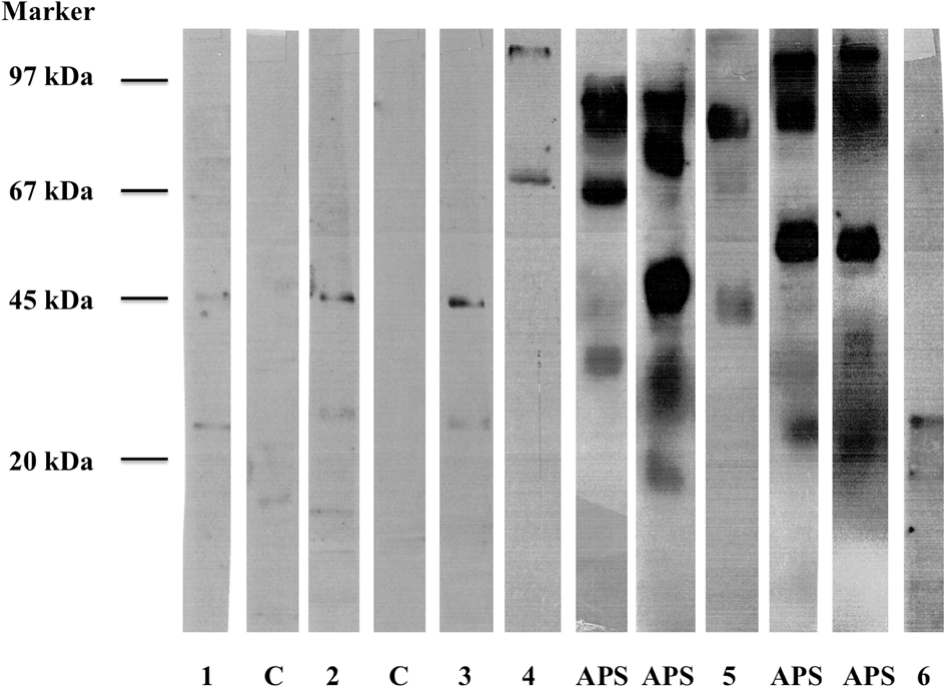
Reactivity of microsomal thyroid antigens with autoantibodies from the sera of the combined pituitary hormone deficiency (CPHD) patients (*lanes* 1–6), healthy controls (C) and APS II patients detected by immunoblotting. Patient 4 and 5 had elevated anti-TPO antibodies concentration in blood (61 and 128 IU/ml, respectively). In all APS II patients anti-TPO antibodies levels were over 2000 IU/ml. Marker: Low Molecular Weight Marker (Amersham-Pharmacia Biotech, Sweden)

Sera from ten patients with the highest binding affinity to M-APA and reactivity to at least three MPAs and ten healthy controls were used to test reactivity against thyroid microsomal autoantigens. Autoantibodies against different microsomal thyroid antigens were demonstrated in six CPHD patients, two controls and all APS II patients. Figure 2 shows the examples of immunoblots using sera from CPHD and APS II patients, and from controls indicating recognized antigens. In CPHD patients reactivity against 72, 68, 45, 26, 24 and 14 kDa was present, while in APS II patients and controls reactivity against poorly defined 68–102 kDa prevailed. RIA method used to estimate the level of autoantibodies demonstrated that low binding was present in five CPHD patients and in two controls, and moderate binding was present in one CPHD patient. In all APS II patients binding was high. The highest binding was demonstrated in cases with reactivity against 72–102 kDa.

## Discussion

In the course of many autoimmune disorders, antibodies present in the serum are reliable markers of the disease. As far as APAs are concerned, it is still unclear whether their appearance is of any diagnostic or prognostic value. So far, a straightforward correlation between the occurrence of these antibodies and the degree of pituitary insufficiency has not been confirmed. This may result from serious methodological problems to identify a specific autoantigen. Therefore, the question as to the actual contribution of autoimmune processes to the primary pituitary disorder pathogenesis is still open (Caturegli 2007). The most popular method to detect the presence of APA is indirect immunofluorescence with the use of human or animal pituitary but the results are often difficult to compare because of different origins of the used pituitary fragments (human—rarely, other primates or rodents) (De Bellis et al. 2003, 2007). Immunoblotting is used less frequently, however it is regarded as a much more specific method, which helps to recognize linear epitopes and may show the presence of many autoantigens (Crock 1998; Karounos et al. 1990). Homogenate of human pituitaries taken from autopsies was used in this method as a substrate. Immunoblotting was demonstrated as a method of antigen identification, e.g. in the case of 49 kDa α-enolase and 68 or 43 kDa pituitary membrane antigens (Bensing et al. 2005; Crock 1998; Nishiki et al. 2001). In our study this method has been chosen to identify pituitary microsomal autoantigens that induce immunoreactivity in patients with childhood-onset combined CPHD. There are only few studies reporting presence of APA in patients with hypopituitarism. Most of these investigations were performed using indirect immunofluorescence and results indicate a presence of APA in 28 % (Stromberg et al. 1998) or 33.3 % (De Bellis et al. 2003) of patients with the growth hormone deficiency.

In our study, considering higher than 2SD of healthy controls M-APA levels as positive, it was demonstrated that sera from all CPHD patients showed low to high immunoreactivity against MPA. Relatively common positivity for M-APA in CPHD might be explained by the fact that the average level of antibodies in controls was low, hence the dispersion of the results was small as well. This observation prompted us to consider the level over 4SD as significant. The autoantibodies with a high binding affinity, which could be considered as a good marker of pituitary autoimmunity, were detected only in 24.2 % of the patients.

To date, the presence of M-APA was previously reported in patients with different pituitary disorders like pituitary tumours before and after radiotherapy, empty sella syndrome and hypopituitarism. These studies revealed reactivity against 58, 60, 67, 68 and 100 kDa antigens (Sawicka et al. 1999). The other studies revealed the presence of M-APA in patients with Hashimoto thyroiditis (60 %), Graves’ disease (72 %), and Addison’s disease (74 %) (Gryczynska and Kosowicz 1991; Gut et al. 2007, 2008). This may indicate that the presence of M-APA in patients with different autoimmune diseases and other pituitary problems is relatively common, but still their role is unclear.

We speculate whether the presence of antibodies targeted against pituitary microsomal antigens results from coexisting defect of the immune system or is only facilitating autoantigen presentation in patients with CPHD (Cline and Radic 2004). Microsomes are small vesicle structures originating from membrane fragments of the Golgi apparatus, endoplasmic reticulum and cell membranes. An example of microsomal autoantigen being a marker of autoimmune process is the TPO (Czarnocka et al. 1985). Our study indicated many different microsomal proteins with immunogenic potential, but the most significant were those with molecular mass of 97, 67, 60, 55 and 36 kDa. In our study, the presence of M-APA at high and moderate level was positively correlated in our study to the duration of the disease, which could be related to ageing autoimmunity or stimulation with persisting antigens population of memory T or B cells. Study on the tissue specificity of the MPA using immunoblots with CPHD and APS II sera that were conducted on the antigens derived from the thyroid gland revealed the immunoreactivity against numerous antigens. We confirmed the immune reaction against 55 and 67–68 kDa thyroid proteins (similar molecular weight to those isolated from the pituitary) in two and one patients, respectively with APS II. The APS II patients also presented high but poorly defined reactivity against 67–102 kDa what may indicate the reaction with TPO and its less or more degraded isoforms. In general, the reaction of the CPHD patients’ sera with thyroid microsomal antigens was weak (apart from one case) and detected reactivity was demonstrated mainly against 24 and 45 kDa proteins. It seems that at least 97, 67, 60 kDa are exclusively limited to the pituitary gland.

Treatment with rhGH in adult patients with a childhood-onset GH deficiency was reported to increase the proportion of CD4–CD8 lymphocytes and shown to induce immune complexes’ formation (Lebl et al. 2000). In our study, however, there was no difference in M-APA levels between treated and untreated patients with rhGH. There was also no difference in M-APA concentration between patients with as well as without genetic background of pituitary disorder.

Interesting results are provided by a simulation based on molecular weight of detected MPA leading to hypothesis: what kind of already known proteins could be those unidentified autoantigens? Examples of proteins having molecular weights approximate to those we have detected and which therefore could be present in the microsomal fraction are listed in Table 5. Some of them already have been described as autoantigens in other autoimmune disorders. These candidate proteins belong mostly to the group of chaperones from the endoplasmic reticulum (ER) and cytosol (like heat shock proteins) or to the Golgi complex antigens (Golgins). Both groups were shown to be involved in autoimmune processes (Routsias and Tzioufas 2006; Stinton et al. 2004), but none of them seem to be exclusively specific for the pituitary. Some proteins are mostly expressed in endocrine tissues and/or the brain. The way in which they become a real immunogen is still under study, but we know that the expression of ER chaperons during cell stress is increased and their translocation to the cytosol and cell membrane occurs. These events may potentiate their immunogenic character. There are different factors causing cell stress such as drugs, infections or thermal shock (Wiersma et al. 2015). Whether these factors (for example common viral infections during childhood) may affect pituitary morphology and function, even many years after incident, remains unclear. Cell death caused by injury, infection or any other factor (e.g. genetic defects) that leads to the increase in apoptotic rate may play a role in the formation of potential autoantigens derived from cytosol and microsomes. An interesting example is the cytochrome P450 2D6, the microsomal autoantigen found to be related to autoimmune hepatitis. This 50/55 kDa (two isoforms) protein is ubiquitously expressed (also in the pituitary) but the autoimmune reaction is present only within the liver. The breaking tolerance to this autoantigen may be caused by an infection with the liver-tropic viruses (Alvarez et al. 1985; Holdener et al. 2008).

**Table 5:** Examples of known proteins whose molecular weights correspond to those of pituitary microsomal autoantigens found in the pituitary microsomal fraction (UniProt database; accessed on http://www.uniprot.org)

Molecular Weight (kDa)	Known corresponding proteins	Expression	Intracellular localization	Reference for reported role in autoimmunity
105	Islet cell antigen 512	Pancreas, brain, pituitary	Secretory granules	(Rabin et al. 1994)
	Tudor domain containing 6 (TDRD6)	Gonads, pituitary	Secretory granules	(Bensing et al. 2007a)
97	Hsp 105 isoforms: alpha and 4	Brain, testis	Cytoplasm	-
	Golgin-97	Ubiquitous	Golgi apparatus	(Griffith et al. 1997)
68	Clathrin interactor 1 (CLINT1)	Ubiquitous	Cytoplasmic vesicles	-
67	Glutamic acid decarboxylase (GAD67)	Endocrine organs	Transport vesicles	(Ali et al. 2011)
	Golgin 67 (GOLGA8B)	Ubiquitous	Golgi apparatus	(Eystathioy et al. 2000)
62	Protein wntless homolog isoform1	Ubiquitous	Golgi apparatus	-
60	Heat shock protein (Hsp 60)	Ubiquitous	Secretory granules	(Kasperkiewicz et al. 2014)
	Golgi resident protein (Gcp 60)	Ubiquitous	ER	-
57	Vesicular inhibitory amino acid transporter (VGAT)	Brain, pituitary	Cytoplasmic vesicles	-
55	Hsp 70 Protein 14	Ubiquitous	Cytoplasm	(Bonaguri et al. 2014)
	Carboxypeptidase E	Ubiquitous	Cytoplasmic vesicles	-
	Cytochrome P450 2D6	Ubiquitous	ER	(Alvarez et al. 1985)
52	Presenilin-1	Ubiquitous	ER	-
	Carbohydrate sulfotransferase 9	Pituitary, trachea	Golgi apparatus	-
45	Golgin-45	Ubiquitous	Golgi apparatus	-
	RAB 6-interacting golgin	Ubiquitous	Golgi apparatus	-
36	Coatomer protein complex	Ubiquitous	Golgi apparatus	-
	Golgi to ER traffic protein 4 homolog	Pituitary and others	Golgi apparatus	-
20	GH 20 kDa isoform	Pituitary	Secretory granules	-
	Carboxypeptidase Q	Ubiquitous	ER, Golgi apparatus	-
18	peptidyl-prolyl cis-trans isomerase (AIP)	Pituitary	Secretory vesicles	-

*ER* endoplasmic reticulum

Autoantibodies are the main marker of autoimmunization. They appear much earlier than the primary symptoms of the disease. In the sera of healthy individuals, certain numbers of natural autoantibodies are present. However, their concentration is mostly very low and therefore of no pathogenic nature (Crock 1998; Lleo et al. 2010).

There is still no answer to the question whether autoimmune processes might be responsible for the development of CPHD or they only modify the course of the disease. Their appearance may be a primary or a secondary factor that influences the development of pituitary alterations, but conceivably the presence of M-APA could be a marker of autoimmune pituitary disorders. Looking back at various studies, we probably should search for a specific combination of markers due to diversity of the pituitary cells’ structure and function. The fact that microsomal autoantigens we have identified have a molecular weight, which is similar to that of, previously described ER proteins, such as chaperons suggests that ER autoantigens might be responsible for the induction of autoimmune reaction against the pituitary.

Our findings clearly point out the necessity to continue more advanced studies on the role of autoimmune processes in combined pituitary hormone deficiency. These should include further identification of already detected autoantigens, their exact immunogenic property and pathogenic nature, but also demonstration of their specificity to pituitary.
